# Deciphering the antimicrobial, antibiofilm and membrane stabilizing synergism of *Mikania scandens* (L.) Willd. leaves and stems substantiation through in vitro and in silico studies

**DOI:** 10.1016/j.btre.2023.e00797

**Published:** 2023-04-16

**Authors:** Nadia Islam Tumpa, Md. Helal Uddin Chowdhury, Ankhy Alamgir Asma

**Affiliations:** a Department of Microbiology, University of Chittagong, Chattogram-4331, Bangladesh; b Ethnobotany and Pharmacognosy Lab, Department of Botany, University of Chittagong, Chattogram-4331, Bangladesh

**Keywords:** Antimicrobial, MIC, MBC, Antibiofilm, Membrane stabilization

## Abstract

Considering the traditional application of *Mikania scandens* (L.) Willd. against wounds and itching. Leaves (MSL) and stems (MSS) were sequentially extracted using solvents petroleum-ether, carbon-tetrachloride, chloroform, ethyl-acetate and ethanol. Disk-diffusion assay revealed the ethyl acetate MSL and MSS extracts were the prominent against ten bacteria, five carbapenem-resistant bacteria and one fungal strains. Subsequent quantitative antimicrobial analysis specified MSL extractives more potent over MSS with lower 1500 and 3500µg/ml MIC and MBC value in both gram-negative and positive bacteria. These sturdiest ethyl-acetate MSL extractives antimicrobial efficiency also fostered fungicidal activity having lower 100µg/ml MFC. Whereat, almost homologous 160–180 min timing noted liken to standard ciprofloxacin susceptibility in both strains, 75% biofilm inhibition at 2×MIC concentration along with 92±0.2% membrane stabilizing activities over synthetic counterparts prospected in preceding standard extractives. Computational molecular docking of MSL compounds supported this findings therefore forego this valuable synergistic insight as antimicrobial agents to efficiently eradicate human infections.

## Highlights

· Multidrug-resistant bacterial infections as well as associated diseases are one of the ever-growing concerns of the modern world.
· *Mikania scandens* like traditional natural affluence of such diseases can be the best alternative.
· Leaves of *Mikania scandens* corroborated its ethnomedicinal infectious utility against a panel of microbes along in inflammation.
· Notably, this same parts comprised exorbitance activity against carbapenem resistant *Pseudomonas aeruginosa* as evident to MIC, MBC and biofilm inhibition.
· This antibacterial and antiinflammatory resilience was further affirmed using computational investigations.

## Graphical abstract

**Figure:**
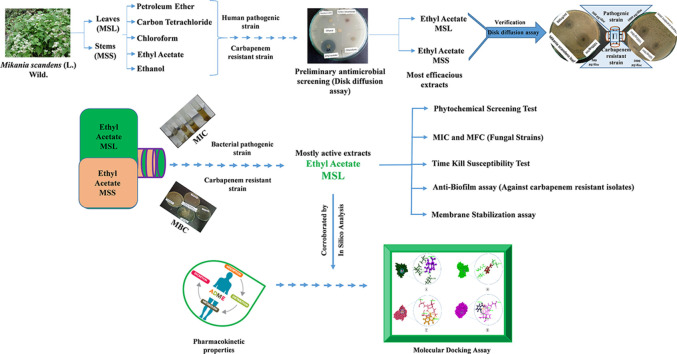

## 1 Introduction

Irresponsible and uncontrolled use of antibiotics resurge the evolution of multi drug resistant (MDR) pathogens which imparts a serious havoc for human races across the globe through hatching plaguing like exigency that culminate the morbidity and mortality rates indicatively [1]. That genesis of MDR meanwhile prospected in Staphylococcus aureus as methicillin resistant S. aureus (MRSA) and pan-resistant Pseudomonas aeruginosa strain emerged major apprehension for its infectivity [2]. Traditional antibiotics' mode of action against these pathogens were inhibition of planktonic bacterial cell growth instead of focusing on the naturally occurring mode of bacterial growth, known as biofilm, which formulated an exopolymeric physical layer on surroundings which resists the pathogens from its unfavorable environment and contributes to recurrent infections likely periodontosis, cystic fibrosis, chronic prostatitis, endocardititis and antibiotic resistance [3]. Over 60% of microbial illnesses may be induced by biofilms, which also account for two-thirds of all bacterial infections in humans [4]. Therefore, inflammatory mediated pathogenic infections is one of the major factor for tumor progression which subsequently aftermath to the apparition of cancer such pathogens likely Helicobacter pylori for gastric cancer, Chlamydia psittaci for ocular lymphomas and Salmonella typhi for hepatobiliary carcinoma [5,6]. On account of Center for Disease Control and Prevention (CDC), around 20000 fatalities worldwide were attributable to pathogenic resistance among the 2 million reported case [7]. By 2050, it is predicted that there would be 10 million more fatalities globally due to antimicrobial resistance, with a treatment price tag of almost $100 trillion [8]. Pharmacological target for the tackling this pathogenic infections, biofilm inhibitory antibiofilm medications should be the subject of intensive study [9]. Various green nonlethal methods for controlling biofilms have been developed recently, but none have yet demonstrated efficacy due to the fact that the new antibiofilm medications' mode of action is substantially less vulnerable to the development of resistance [10]. The exploration of medicinal plants utilizing cutting-edge technology is currently being reevaluated as safe conventional treatments for pathogenic infections and as a practical method for identifying novel antimicrobial bioactive molecules to address this pervasive public health issues [11]. Researchers are seeking for natural compounds that have antibacterial action and work in synergy with currently available antimicrobial therapies since microbes that are resistant to conventional treatments are becoming more prevalent. As a result, herbal remedies are emerging as a promising alternative to conventional infection treatment.

*Mikania scandens* (L.) (MS) is a twining plant that can be found growing as a weed throughout the country and locally known as “Jarmany lota” in Bangladesh [12]. Intersecting analysis of various literature unveiled that leaf of this plant traditionally used in several part of the globe as remedial of wounds, bruise, itching and headache [13], [14], [15], [16]. This pain modulation affluence therefore scientifically verified [17] along disclosing antioxidant [18], anti-inflammatory [19], antidiabetic [20], thrombolytic [21] and neuropharmacological [22] potency of MS. Mikanolide, a sesquiterpene lactone metabolite, has been isolated from this MS plant so far [23].

In this study, the information gleaned herein is used as baseline to define the MS plants' leaves and stems antimicrobial and antbiofilm activities as well as their synergism with regard to anti-inflammatory potentialities using a range of in vitro and in silico screening methodologies.

## 2 Materials and methods

### 2.1 Collection of plant materials

Fresh mature plant part stems and leaves of *Mikania scandens* (L.) were collected from the Chittagong University campus. Thereafter, this plant was authenticated by one of the countries prominent taxonomist Prof. Dr. Shaikh Bokhtear Uddin, Department of Botany at the University of Chittagong. Subsequently, following standard herbarium techniques one of the voucher specimen had been deposited in the Herbarium of Chittagong University (HCU) for future reference. The samples (leaves, stems) were cut into small pieces (1-2cm), washed thoroughly using distilled water, air-dried at room temperature, and pulverized to fine powder mechanically. During this precautions taken to avoid admixture of each of the plant parts.

### 2.2 Preparation of extracts

The fine powdered of each of the plant parts (leaves and stems) samples were sequentially extracted with polar and non-polar solvents based on solvents polarity index (Petroleum ether, Carbon tetrachloride, Chloroform, Ethyl acetate, and Ethanol). Whereat 200g powder was amalgamated with 500 ml solvents for extraction in a dark vessel and kept it there for 5 days with manual application of gentle shaking. Then, the extracts were filtered using Whitman filter paper and concentrated at low temperatures (40-50°C) by a rotary vacuum evaporator. Thus, the extract obtained was termed crude extract. The crude extract of each sample was weighed and calculated the yield percentage of each sample as follows:

$$\text{Extract}\text{yield}\%=\left(W_{1}/W_{2}\right)\times 100$$

Here, W_1_= Net weight of powder in grams after extraction.
And W_2_= Total weight of wood powder in grams taken for extraction.

### 2.3 Chemicals, reagents and equipment

Petroleum ether, Carbon tetrachloride, Chloroform, Ethyl acetate, and Ethanol culled from Sigma Chemicals Co. (St. Louis, MO, USA). Ciprofloxacin, and Diclofenac Sodium retrieved from ACI Ltd in Sonargaon, Bangladesh as well as isosaline collected from Popular Pharmaceuticals Ltd. in West Panthapath, Dhaka, Bangladesh. The chemical from Merck (Darmstadt, Germany) namely Ammonium molybdate, ferric chloride (FeCl_3_), sodium carbonate (Na_2_CO_3_), hydrogen peroxide (H_2_O_2_), hydrochloric acid (HCl), sulfuric acid (H_2_SO_4_), potassium acetate, phosphate buffer, and dimethyl sulfoxide (DMSO) were acquired. UV-VIS Spectrophotometer utilized to signify the changes in absorbance (UVmini-1240, Shimadzu, Japan).

### 2.4 Test organisms

All of the microorganisms used in the tests were taken from culture collection stocks maintained by the Molecular Biology Lab of Department of Microbiology at the University of Chittagong

#### 2.4.1 Bacterial strains

The bacterial organisms utilized for the antimicrobial potency determination against a set of organisms were listed below-

Gram negative (*Escherichia coli* ATCC25923, *Pseudomonas aeruginosa* ATCC8027, *Salmonella abony* NCTC6017, *Klebsiella pneumoniae* ATCC13883, *Vibrio cholerae* AE14748, *Shigella dysenteriae* AE14612, *Acinetobacter baumannii* ATCC17978) and gram positive (*Bacillus cereus* BTCC19, *Bacillus subtilis* ATCC6633 and *Staphylococcus aureus* ATCC6538)*.* Besides these, five carbapenem resistant pathogens isolated from burn wound infections were tested these are *Klebsiella pneumoniae* (KP_5_), *Pseudomonas aeruginosa* (PA_3_), *Escherichia coli* (EC_5_), *Acinetobacter baumannii* (AB_5_), and *Staphylococcus aureus* (SA_3_).

#### 2.4.2 Fungal strains

The fungus *Candida albicans* ATCC10231, *Aspergillus niger* ATCC16404*, Aspergillus fumigatus* and *Penicillum sp.* were culled for antifungal properties evaluation of MS crude extracts of several solvents.

### 2.5 Determination of antimicrobial activity

The antibacterial and antifungal activities of the plant extracts were done by disc diffusion method and poisoned food technique respectively [24,25]. Wherein, Nutrient agar (NA) was used to culture the test bacteria; Muller-Hinton Agar (MHA) medium was used for disc diffusion method and Potato Dextrose Agar (PDA) medium was used for the culture of fungi with the exception of *Candida albicans*, whose nutritional needs and plate development are very similar to those of bacteria*.* A 4 mm size Whatman filter paper disc composed 10 µl of each plant extract integrated on the LB media plates for bacteria with having 1.6 × 10^7^ CFU/ml organismal strain and PDA plates with 1.7 × 10^8^ CFU/ml fungal strain. Thereby, those bacterial and fungal plates were incubated at 37° C and room temperature for 24h following respectively after 4h fridge storage at 4°C for dissemination of extracts.

### 2.6 Phytochemical analysis of mostly active extracts

The extracts were tested for significant phytochemicals according to recognized procedures. Several chemical assays utilizing accepted techniques were used to screen the aforementioned medicinal plant extracts for diverse phytochemical components [26,27].

### 2.7 Determination of MIC and MBC

A two fold macro-broth dilution method in accordance with Clinical and Laboratorial Standards Institute was employed to ascertain the minimum inhibitory concentration (MIC), minimum bactericidal concentration (MBC), and minimum fungicidal concentration (MFC) of the ethyl acetate extracts of MSL and MSS [28]. Each tube was inoculated with 1 ml of diluted bacterial (1.6 × 10^7^ CFU/ml) and fungal (1.7 × 10^8^ CFU/ml) suspension, each of which included 1 ml of LB (for bacteria) and Sabouraud (for fungi) broth, as well as a variety of test materials with concentrations ranging from 500 to 2500 µg/ml. Following that, both tubes had a 24h incubation period—for bacteria, at 37°C, and for fungus, at room temperature.

A suspension aliquot of 0.5 ml was placed into pre-sterilized petri dishes with MHA and PDA medium in order to explore the potential MBC, and MFC of antimicrobial agent in broth culture tubes where the microbial growth was not visible. After that, the plates were incubated for 24h at 37°C for bacteria and room temperature for fungal inoculum in order to observe the microbial growth. MBC and MFC were defined as the maximum dilution at which at least 99% of bacteria were inhibited. Testing was undertaken in triplicate for each extract concentrations.

### 2.8 Time kill bacterial susceptibility to MSL and MSS extracts

Time-kill bacterial sensitivity to extracts was examined, according to Ajiboye et al (2016) description [29]. In Luria Bertani (LB) medium, bacterial cells were cultured during the night. Afterward, the cells were collected by centrifugation and resuspended in 50 mL of new LB media to produce an OD600 = 0.1 in a 250 mL conical flask. After that, the cells were grown aerobically at 37°C in a shaker incubator. 15ml of culture were divided among 3 conical flasks at mid-log phase (OD600 = 0.5). After that, an antibiotic (ciprofloxacin) was added as a positive control, 0.5% dimethyl sulfoxide was added as a negative control, and crude extract of the tested organisms at MBC concentration. After that, the mixes were incubated for 3 hours at 37°C.

Every 20 minutes for the first three hours of incubation, the incubation mixture's absorbance was measured at 600 nm. Samples of the control cultures and the extract-treated cultures were taken for colony formation at intervals of 0, 20, 40, 60, 80, 100, 120, 140, 160, and 180 min, respectively, and centrifuged to collect the cells as a pellet. After being thoroughly cleaned, the cells were diluted with 0.9% NaCl, combined with molten soft LB-agar at 42°C, and then put onto agar plates with solid LB-agar. The plates were then housed in an incubator at 37 °C. Colonies were counted 24 hours later.

### 2.9 Anti-biofilm activity test

The crystal violet staining technique, as significantly modified by Christensen et al. (1985), was used to assess the impact of test extracts on biofilm development [30]. Bacterial cultures were performed using LB broth medium. Each test tube received 0.5ml of plant extract (0.5× MIC, 1×MIC, and 2×MIC). The negative control was considered to be equal amounts of water. Each test tube received a final volume of 1ml after 0.5ml of bacterial suspension (from a 24-hour culture) was added. As an extra nutrition, sterile LB was also supplied to each tube. Following that, the test tubes were kept at 37°C for three days to facilitate the cell adherence.

The test tubes were cleaned five times with sterile distilled water after incubation to get rid of any bacterial cells that were loosely adhered. Following drying, the tubes were stained with 1% crystal violet. The tubes were rinsed to remove extra stain after 15 minutes. The test tube was once again destaining with 2 ml of 90% ethanol. At 530 nm, the destained solution's absorbance was quantified. Percentage of biofilm inhibition was measured using the following equation:

$$\%\text{of}\text{inhibition}=100-\left[\left\{\left(OD_{530}\text{nm}\text{of}\text{experimental}\right)/OD_{530}\text{nm}\text{of}\text{control})\right\}\times 100\right]$$

### 2.10 Membrane stabilization test

The techniques of hemolysis caused by a hypotonic solution were used to scrutinize the membrane stability of extracts in vitro anti-inflammatory effectiveness using the human red blood cell (HRBC) membrane stabilization procedure of Vuna & Botting (1995) [31].

Six individuals provided their fresh whole blood, which was then combined with an equivalent amount of sterilized Alsever solution and centrifuged at 3000 rpm for 10 minutes. The packed cells were then washed three times with isosaline before being made into a 10% v/v suspension using isosaline. Hypotonic solution (5 ml) and erythrocyte suspension (0. 5 ml) were combined. Next, the extractives (25–200 µg/ml) and a conventional medication (Diclofenac sodium) were applied to certain samples and not to others. Extracts and drugs were not introduced to the control group. Following a 10-minute incubation period, the mixture was centrifuged at 3000 rpm for 20 minutes to determine the amount of hemoglobin. After the supernatant was decanted, the hemoglobin content was calculated at 560 nm.

$$\%\text{of}\text{hemolysis}=\frac{\text{The}\text{optical}\text{density}\text{of}\text{test}\text{samples}}{\text{The}\text{optical}\text{density}\text{of}\text{the}\text{control}}\times 100$$

### 2.11 Acquisition of compounds and theirs pharmacokinetic properties

From *M. scandens* leaves, the literature review compiled 23 (Table 8) previously discovered active components [12]. With the aid of PubChem (https://pubchem.ncbi.nlm.nih.gov/) and SWISSADME (http://www.swissadme.ch/), the information about the gathered compounds and their drug similarity characteristics were retrieved and computed. SWISSADME filtered the drug-likeness distinctive features of the active components from *M. scandens* leaves using compound canonical SMILES from the PubChem database based on Lipinski's rule of five [32].

### 2.12 Ligands and targets modulation for molecular docking

Potent ligands discovered through drug likeness criteria, and the reference drug ciprofloxacin and sertaconazoles were all downloaded in .sdf format from the PubChem chemical library. Additionally, the LigPrep tools prepared the compounds for molecular docking investigations that embedded to Schrodinger's suite-Maestro v12.5 using the procedures we previously outlined [33]. The crystal structures of each target protein were gathered from the RCSB Protein Data Bank in order to optimize and minimize the each structures using another integrated tools protein preparation wizard. With OPL3S force field, all charges and bond orders were assigned, heavy atoms were allocated hydrogen, selenomethionine was substituted out for methionine, water was removed [34].

### 2.13 Standard precision molecular docking by Glide

To create receptor grids and carry out flexible molecular docking tests, Glide, an add-on for Schrodinger Suite-Maestro version 12.5, was utilized [35]. The force field of OPLS3 was used to generate a grid for each protein using the default settings of van der Waals scaling factor 1.00 and charge cut-off value 0.25. A cubic box of 14 Å × 14 Å × 14 Å size was generated over the active site (co-crystallize ligand site) pockets of each protein. The best scoring pose and docking value about each ligand were individually recorded throughout the docking assays, whom were completed using Glide's Standard Precision (SP) scoring system.

## 3 Results and discussions

### 3.1 Yielding percentage of MSL and MSS extractives

The yielding percentage of extractives by different solvents Petroleum ether, carbon tetrachloride, chloroform, ethyl acetate and ethanol were 1.7%, 4%, 2.4%, 14.5% and 23.3% respectively from MSL and 0.9%, 5%, 1.65%, 8.9% and 27.5% respectively from MSS. That clearly indicated that the quantity of polar molecules was far higher than that of non-polar elements.

### 3.2 Qualitative antimicrobial activity test of MSL and MSS extractives

Variable non-polar (petroleum ether, carbon tetrachloride, chloroform) and polar (ethyl acetate and ethanol) solvent extracts from *Mikania scandens* stems (MSS) and leaves (MSL) had been exerted to ordain the antimicrobial efficiency against eleven human pathogenic organisms and five carbapenem-resistant isolates from burn wound infections in disk diffusion assay. The most practical approach for determining whether microbes are susceptible or resistant to different antimicrobial agents is the disc-diffusion method (DDM); nevertheless, the accuracy of this method depends on the upkeep of standard operating protocols [36]. Whereas, by the use of MSL extracts against bacterial pathogen, non-polar solvents showed an inhibitory zone of 9 to 23 mm that was somewhat larger than the polar extracts' 8 to 22 mm zone. On them, the petroleum ether and ethyl acetate extracts were the foremost one toward *K. pneumoniae* and *P. aeruginosa* which explicitly parallelism to standards ciprofloxacin effects. This ethyl acetate extracts supremacy therefore observed in aerial part extract of this same plants constructing the highest 9.37 mm inhibitory zone which articulately preceded by present studies [37]. Likewise, this research emphasized the furthest zone of inhibition that over some plant extracts displayed [38], [39], [40]. In the case of fungi, Ethyl acetate extracts from polar solvents conferred a much larger 24 ± 1.6 mm inhibitory zone against fungus *Candida albicans* when compared to non-polar carbon tetrachlorides 18 ± 1.25 mm zone. That furthest zone diameter whither pointedly over than sertaconazoles 22 ± 1.7 mm zone in diameter (Table 1). Contrariwise, *Sh. dysnteriae* resistant to all the extracts of MEL. Other hand, MSS non-polar extracts exhibited lower sensitivity against five bacterial (out of 10) and one fungal pathogens with subordinate zone of inhibition exception to *E. coli*’s 20 ± 1.3 mm zone by petroleum ether. Conversely polar ethyl extracts of stem consistent to its leaf potentialities having notable 20 ± 1.7 mm and 18 ± 1.3 mm zone in both bacteria and fungi. Noticeably, chloroform extracts of leaf, carbon tetrachloride and ethanol extracts of stems asserted scanty antibacterial susceptibility, virtually showing resistance to those pathogens infections (Table 2). This existence of variation in antimicrobial susceptibility might be due to the presence of diverse amount of phenolic and flavonoid compounds in plants that easily accessible to permeability of the cell wall of bacteria for disrupting the cells [41,42]. Preliminary phytochemical screening of both MSL and MSS certified that existence (Table 3), whilst literature however subsistence the 456.3 ± 0.08 mg of GA/g phenolic and 672.8 ± 0.76 mg of CA/g flavonoidic contents in MS [43]. That pattern of resistance also sustained against carbapenem resistant clinical isolates pathogens with chloroform extracts of MSL, and carbon tetrachloride and chloroform extracts of MSS. But ethyl acetate of both MSL and MSS substantially had greater extent of efficacy against all of five isolates specifically *S. aureus* with ethyl acetate MSL (20 ± 1.6 mm zone) and ethanol MSS (16 ± 1.5 mm zone) (Tables 1 and 2). Relatively, the respective plant lineages*, M. hirsutissima* [44]and *M. cordata* [45], maintained their potency against *S. aureus*. In both plant parts ethyl acetate extracts demonstrated supreme therapeutic efficacy; hence, envisaged as benchmark extracts for further studies.

**Table 1:** Antimicrobial efficacy of five extracts of *Mikania scandens* leaf (MSL) against human pathogens.

Test organisms	Zone of inhibition (mm in diameter)					
	Petroleum ether	Carbon tetrachloride	Chloroform	Ethyl acetate	Ethanol	Ciprofloxacin/ Sertaconazole
*Escherichia coli* ATCC2593	-	11.3±1.5	-	20±1	-	24.7±1.2
*Klebsiella pneumoniae* ATCC13883	**23±1**	15±1	-	-	10±0.5	21.3±2.2
*Staphylococcus aureus* ATCC6538	-	15.6±0.5	11±0.5	14.4±1.6	11±0.6	20.3±1.9
*Salmonella abony* NCTC6017	15.5±0.5	13.2±1.1	-	-	-	18.7±1
*Acinetobacter baumannii* ATCC17978	9.25±0.5	**15**±1.4	-	13±0.6	8±0.5	13.8±1.1
*Bacillus cereus* BTCC19	-	9±0.5	-	20±1.3	15±1.5	25±2.2
*Bacillus subtilis* ATCC6633	-	11.6±0.7	10±0.5	-	-	27.8±2.6
*Pseudomonas aeruginosa* ATCC8027	12.3±1.3	10±0.3	-	22±1.5	20.4±1.3	22.4±1.5
*Vibrio cholerae* AE14748	-	17±0.8	-	12±1.1	12.3±1.5	20.3±2.1
*Shigella dysenteriae* AE14612	-	-	-	-	-	29±2.2
*Candida albicans* ATCC10231*	15.7±1.4	18±1.25	-	24±1.6	-	22±1.7
**Carbapenem resistant**						-
*Escherichia coli* (EC_5_)	8±0.3	15±1.3	-	17±1	-	-
*Klebsiella pneumoniae* (KP_5_)	-	-	-	12±1.5	10±1	-
*Staphylococcus aureus* (SA_3_)	10±0.7	-	-	20±1.6	14.5±1.6	-
*Pseudomonas aeruginosa* (PA_3_)	-	9±1	-	9.4±0.3	-	-
*Acinetobacter baumannii* (AB_5_)	-	15±1.8	-	12±1.3	-	

Note: 4mm in diameter paper disc soaked with different extracts; here, bold indicated highest zone of inhibition and (-) means no inhibition. *Fungal pathogen.

**Table 2:** Antimicrobial activity of five extracts of *Mikania scandens* stem (MSS) against human pathogens

Test organisms	Zone of inhibition (mm in diameter)					
	Petroleum ether	Carbon tetrachloride	Chloro form	Ethyl acetate	Ethanol	Ciprofloxacin/ Sertaconazole
*Escherichia coli* ATCC2593	20±1.3	-	-	-	-	24.7±1.2
*Klebsiella pneumoniae* ATCC13883	-	-	-	10.3±0.3	-	21.3±2.2
*Staphylococcus aureus* ATCC6538	-	-	7±0.5	15±1.3	6±0.3	20.3±1.9
*Salmonella abony* NCTC6017	-	-	-	10±1	-	18.7±1
*Acinetobacter baumannii* ATCC17978	-	-	-	13±2.1	-	13.8±1.1
*Bacillus cereus* BTCC19	10±1	12±0.7	12.3±1.4	17±2.2	-	25±2.2
*Bacillus subtilis* ATCC6633	-	10±0.3	7±1	20±1.7	6±0.3	27.8±2.6
*Pseudomonas aeruginosa* ATCC8027	16.3±0.7	-	-	-	-	22.4±1.5
*Vibrio cholerae* AE14748	-	-	-	-	-	20.3±2.1
*Shigella dysenteriae* AE14612	-	-	7±0.3	12.5±1.5	10±1	29±2.2
*Candida albicans* ATCC10231*	-	7.5±1.5	9±1	18±1.3	-	22±1.7
**Carbapenem resistant**						
*Escherichia coli* (EC_5_)	-	-	-	10±1.3	-	-
*Klebsiella pneumoniae* (KP_5_)	10±1	-	-	6±0.5	-	-
*Staphylococcus aureus* (SA_3_)	7±0.5	-	-	-	16±1.5	-
*Pseudomonas aeruginosa* (PA_3_)	-	-	-	-	-	-
*Acinetobacter baumannii* (AB_5_)	-	-	-	15±1.3	-	-

Note: 4 mm in diameter paper disc soaked with different extracts; here, bold indicated highest zone of inhibition and (-) means no inhibition. * Fungal pathogen.

**Table 3:** Qualitative phytochemical screening of MSL and MSS extracts

Components	MSL	MSS
Saponin	+	+
Tannin	+	+
Alkaloid	-	+
Phenols	+	+
Flavonoids	+	+
Anthocyanin	+	+
Betacyanin	-	-
Carbohydrate	-	-
Fats and oil	-	-
Steroid	-	+
Phytosteroid	-	-

**Fig. 1:**
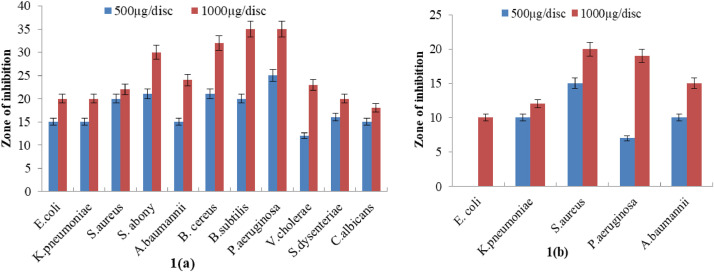
Antimicrobial activity of ethyl acetate *Mikania scandens* leaves (MSL) extracts against (a) human pathogenic microrganisms and (b) carbapenem-resistant pathogens at both 500µg/disc

**Fig. 2:**
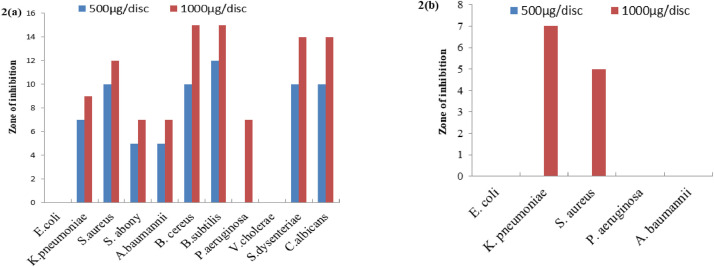
Antimicrobial activity of ethyl acetate *Mikania scandens* stems (MSS) extracts against (a) human pathogenic organisms and (b) Carbapenem-resistant pathogens at the concentration of 500 µg/disc and 1000 µg/disc.

This standard ethyl acetate extracts of MSL (Fig. 1) and MSS (Fig. 2) further evaluated for antimicrobial susceptibility at two different concentrations of 500 µg/disc and 1000 µg/disc. MSL at both concentrations (at 1000 µg/disc= 20 – 35 mm and at 500 µg/disc= 12 – 23 mm inhibitory zone) showed dose dependent antimicrobial activity for all organisms tested here including carbapenenm resistant microorganisms except *E. coli* at 500 µg/disc concentration. This extracts notable affluence potency against *S. aureus* meanwhile preserved in here with 20 mm inhibition zone at 1000 µg/disc in both types of strains. Whereas, comparatively less effectiveness of ethyl acetate MSS than its MSL, correlated the precedent findings containing an array of 7 – 15 mm inhibition zone at the greatest concentration along resistance to *E. coli* and *V. cholera*. This contextual fact therewithal seen against carbapenem resistant microbes with portraying almost entire panel of microbial resistance.

Table 4 displays the percentage of the fungal radial mycelial growth that was inhibited. At the concentration of 50 and 100 µg/ml, the ethyl acetate MSL extract may completely inhibit the *Penecillium* sp., *A. niger* that was cabbalistically outdo the standard sertaconozols inhibition. Conversely, *A. fumigatus* exhibited less inhibition than other species. This new threshold for maximal inhibitory doses of 100 µg/ml surpasses the previous studies on the antifungal properties of plant extracts [46], [47], [48].

**Table 4:** Antifungal growth of prominent plant extracts ethyl acetate MSL at different concentrations.

Tested fungi	Percentage (%) of inhibition			
	25µg/ml	50µg/ml	100µg/ml	Sertaconazol (3 mg/ml)
*Aspergillus niger* ATCC16404	59.21 ± 1.6	87.5 ± 1.15	100 ± 0	88.2 ± 1.3
*Aspergillus fumigatus* (ATCC46645)	48.37 ± 1.2	54.32 ± 0.87	60 ± 0.45	89.76 ± 0.75
*Penecillium* chrysogenum. (ATCC11709)	88.76 ± 0.31	100 ± 0	100 ± 0	86.45 ± 1.6

### 3.3 Quantitative antimicrobial evaluation of MSL and MSS extractives: Determination of MIC, MBC, and MFC

Bacteriostatic (MIC) and bactericidal (MBC) effectiveness of ethyl acetate extracts of MSL and MSS signified adopting macro-broth dilution method. MSL ethyl acetate extractives markedly inhibit the growth (MIC= 1500 – 2000 µg/ml) and death (MBC= 3000 – 5000 µg/ml) of bacteria against all panel of microorganisms unlike to MSS extracts which resistance to *E. coli, S. aureus, A. baumannii* and *Sh. dysenteriae* at both characteristic levels along not bactericidal to *K. pneumoniae* and *B. cereus* (Table 6). Noticeable lower MIC (≤2000 µg/ml) with MBC (≤4000 µg/ml) value detected in *E.coli, S. aureus, A. baumannii, B. cereus, B. subtilis, V. cholera. P. aeruginosa* and *Sh. dysenteriae* with MSL extractives (Table 5). MBC/MIC ration meanwhile substantiated those strains signature bactericidal effects having the ratio lower ≤4 threshold value where exceeding this value considering bacteriostatic phase [49]. Similar comparable findings for *B. cereus* meanwhile reported in another studies based on the ethyl acetate extracts of MS aerial parts recording MIC and MBC value within 0.38 to 6 mg/ml wherein our studies outdo the *S. aureus, E. coli* and *B. subtilis* value [37]. Two of current plants generic lineages *M. laevigata* and *M. glomerata*’s further substantiated its antibacterial action whereat non-polar solvent hexane extract defined bacterial growth inhibition and death at MIC of 12.5 to 100 mg/ml and MBC of 25 to 400 mg/ml concentrations respectively as indicative of lower polarity bioactive substances which is opposed to current findings [50]. This positive reactiveness of this extracts might be attributed through ATP hydrolysis, coagulation of cytoplasm, altering proton motive force, inhibiting DNA gyrase, protein synthesis, and cell wall permeability [51] and the difference in the MIC and MBC owing to variation in phytoconstituents nature [52]. A number of plant derived alkaloids and phenolic substances comprehensively picturesque theirs antimicrobial robustness restraining the fasten efflux of possible drug candidates that raising the permeability of bacterial cell wall to reach the intrabacterial cytoplasm so that refraining of the ribosomal protein synthesis takes place [53]. Another report also hypothesized the involvement of polyphenols (phenolics, tannins, flavonoids) in the cell membrane and cell wall through forming hydrogen bond within hydroxyl group and enzyme penetrating into cell and doing coagulation of cellular contents [54]. Notwithstanding that bacterial structural alterations of efflux and influx system for resistance documented in *S. aureus, P. aeruginosa* and *A. baumannii* [55,56]. Importantly, this same ethyl acetate MSL extracts disclosed almost considerable eptitude against both gram positive and negative bacteria opposed to conventional plant extracts resistance towards gram-negative bacteria due to hydrophilic cover over peptidoglycan which accountable for easy accessible of plant extractives in gram positive bacteria [57].

**Table 5:** MIC and MBC of prominent ethyl acetate MSL extract against human pathogenic bacteria.

Name of bacteria	Bacterial growth in LB broth (concentration µg/ml)					MIC µg/ml	MBC µg/ml	MBC/MIC
	500	1000	1500	2000	2500			
*Escherichia coli* ATCC2593	+	+	-	-	-	1500	4000	2.67
*Klebsiella pneumoniae* ATCC13883	+	+	+	-	-	2000	5000	2.5
*Staphylococcus aureus* ATCC6538	+	+	+	-	-	2000	4000	2
*Salmonella abony* NCTC6017	+	+	+	-	-	2000	5000	2.5
*Acinetobacter baumannii* ATCC17978	+	+	+	-	-	2000	4000	2
*Bacillus cereus* BTCC19	+	+	-	-	-	1500	3500	2.33
*Bacillus subtilis* ATCC6633	+	+	-	-	-	1500	3500	2.33
*Pseudomonas aeruginosa* ATCC8027	+	+	-	-	-	1500	4000	2.67
*Vibrio cholerae* AE14748	+	+	-	-	-	1500	3500	2.33
*Shigella dysenteriae* AE14612	+	+	-	-	-	1500	4000	2.67
**Carbapenem resistant**								
*Escherichia coli* (EC_5_)	+	+	+	+	+	ND	ND	ND
*Klebsiella pneumoniae* (KP_5_)	+	+	+	-	-	2000	5000	2.5
*Staphylococcus aureus* (SA_3_)	+	+	+	+	+	ND	ND	ND
*Pseudomonas aeruginosa* (PA_3_)	+	+	+	+	-	2500	5000	2
*Acinetobacter baumannii* (AB_5_)	+	+	+	+	+	ND	ND	ND

Note: (+) indicates the presence of growth and (-) means the absence of growth and ND means not detected up to 2500 µg/ml in MIC and MBC was not found up to 5000 µg/ml.

**Table 6:** MIC and MBC of ethyl acetate MSS extract against human pathogenic bacteria.

Name of bacteria	Bacterial growth in LB broth (concentration µg/ml)					MIC µg/ml	MBC µg/ml	MBC/MIC
	500	1000	1500	2000	2500			
*Escherichia coli* ATCC2593	+	+	+	+	+	ND	ND	ND
*Klebsiella pneumoniae* ATCC13883	+	+	+	+	-	2500	ND	ND
*Staphylococcus aureus* ATCC6538	+	+	+	+	+	ND	ND	ND
*Salmonella abony* NCTC6017	+	+	+	-	-	2000	5000	2.5
*Acinetobacter baumannii* ATCC17978	+	+	+	+	+	ND	ND	ND
*Bacillus cereus* BTCC19	+	+	+	+	-	2500	ND	ND
*Bacillus subtilis* ATCC6633	+	+	+	-	-	2000	5000	2.5
*Pseudomonas aeruginosa* ATCC8027	+	+	+	+	-	2500	5000	2
*Vibrio cholerae* AE14748	+	+	+	+	-	2500	ND	ND
*Shigella dysenteriae* AE14612	+	+	+	+	+	ND	ND	ND
**Carbapenem resistant**								
*Escherichia coli* (EC_5_)	+	+	+	+	+	ND	ND	ND
*Klebsiella pneumoniae* (KP_5_)	+	+	+	+	+	ND	ND	ND
*Staphylococcus aureus* (SA_3_)	+	+	+	+	+	ND	ND	ND
*Pseudomonas aeruginosa* (PA_3_)	+	+	+	+	+	ND	ND	ND
*Acinetobacter baumannii* (AB_5_)	+	+	+	+	-	2500	ND	ND

Note: (+) indicates the presence of growth and (-) means the absence of growth and ND means not detected up to 2500 µg/ml in MIC and MBC was not found up to 5000 µg/ml.

Conjointly, resistance to carbapenem workable by mediating β-lactamase and hydrolytic enzymes which each molecules capable of hydrolyzing 10^3^ β-lactam ring per second (drug substance/s) resultant to 100 million molecules elimination in each second with 10^5^ enzymes [58,59]. On those resistant pathogens, ethyl acetate MSL extracts showed dominant sensitivity effect of bacteriostatic and bactericidal with two gram-negative (*P. aeruginosa* and *A. baumannii*) carbapenem resistant medical isolates composing 2000 – 5000 µg/ml concentrations of MIC and MBC along MBC/MIC ratio between 2 – 2.5 clarify theirs bactericidal notion of the extracts (Table 5). Whither, MSS posed only bacteriostatic potential against *A. baumannii* at 2000 µg/ml and persist resistance to other bacterial strains at both MIC and MBC levels. That forcible feasibility against gram-negative bacteria meantime concurrent to antecedent findings. This homologous dominancy also found in one of its generic predecessor *M. micrantha* leaves whereat deoxymikanolide isolated from that plant part exhibited the lowest MIC and MBC value of 62.5 and 125 mg/L respectively against *Xanthomonas campestris* pv. *vesicatoria* and *Xanthomonas campestris* pv. *citri* [60]. Presumably, inhibition of enzymatic modulation and degradation of drug molecules, and probable inhibitors of major facilitator superfamily of efflux pump (EP) tangibly RND (resistance-nodulation division) superfamily of gram-negative bacteria were the functional mode for this MSL extract as per literature reported about carbapenem resistance mechanistic target attribution [61,62].

From Table 7, it was found that the minimum concentrations needed for the fungicidal activity of MSL ethyl acetate extract were 100 µg/ml against *Penicillium spp.* and 200µg/ml against *A. niger* and *A. fumigatus*. However, the MIC values were 50 µg/ml for *Penicillium spp.* and 100 µg/ml for *A. niger* and *A. fumigatus*. Theirs, MFC/MIC proportion was also within 2 as indicative of strong microbiocidal activities. Moreover, Siddiqui et al. (2013) found that the ethyl acetate extract of MSL revealed effective antifungal activity against *F. oxysporum, P. graminicola* and *R. solani* with MIC values of 250-500 µg/ml on phytopathogenic fungi [12].

**Table 7:** MIC and MFC of prominent ethyl acetate MSL extract against fungi.

Name of fungi	Fungal growth in Sabouraud broth (concentration µg/ml)					MIC µg/ml	MFC µg/ml	MFC/MIC
	25	50	100	200	400			
*Aspergillus niger*	+	+	-	-	-	100	200	2
*Aspergillus fumigatus*	+	+	+	+	-	100	200	2
*Penicillium spp.*	+	-	-	-	-	**50**	**100**	**2**

Note: (+) indicates the presence of growth and (-) means the absence of growth. Here bold indicates the lowest MIC and MFC value.

### 3.4 Time-kill bacterial susceptibility with MSL and MSS extractives

One gram negative and gram positive bacteria, namely *E. coli* and *B. cereus*, respectively, were chosen for the time kill sensitivity assay on the basis of ≤4 MBC/MIC ratio with the most efficient ethyl acetate MSL extracts.

**Fig. 3:**
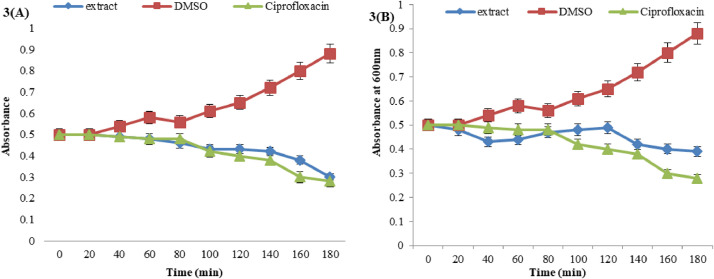
Optical densities of (a) *Escherichia coli* and (b) *Bacillus cereus* exposed to standard ethyl acetate MSL extract at variable times where exposure of extracts showing the absorbance decreasing after an interval of time that sensitive to microbes

When compared to bacteria that were just exposed to DMSO, the absorbance was lowered when *E. coli* and *B. cereus* were treated with an ethyl acetate extract of MSL at bacterial MBC concentration. Similar changes in these bacterial cells' absorbance were brought about by the reference antibiotic Ciprofloxacin. A bacterial cell can be killed, as this study's results shown, with the passage of time. Thus according to Fig. 3A, ethyl acetate MSL extract reduced bacterial cell absorbance at 0.30 almost equivalent to reference drugs absorbance rate and nearly killed *E. coli* in the period of 160 to 180 minutes. It was discovered in Fig. 3B that extracts could not completely kill bacterial cells at the same rate as antibiotics alone to kill gram-positive *B. cereus* in 180 minutes. The cause of their presence of a thick peptidoglycan coating may take a long time to eradicate. This noteworthy antibacterial capacity was investigated against gram-negative bacteria that were atypical for plant extractives to have microbiocidal affluence, this large response was indeed compatible with quantitative antimicrobial screenings of ongoing investigations. In a study by Ajiboye et al. using a similar time-kill susceptibility experiment aligned with our findings, the researchers noted that the extract lowered absorbance relative to bacterial cells treated using simply DMSO [63]. *Euclea crispa* ethyl acetate leaf extracts elucidated complete inhibition of cells in 120 min against gram negative *K. pneumoniae* at 2×MIC concentrations [64].

### 3.5 Antibiofilm properties of MSL and MSS extractives

**Fig. 4:**
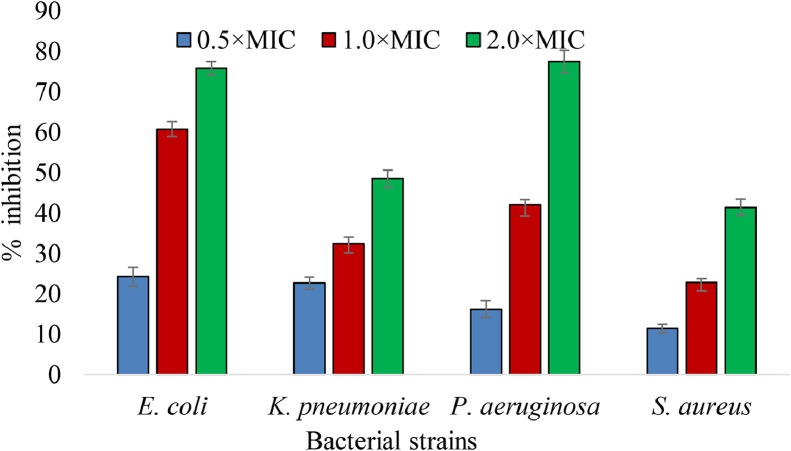
Antibiofilm activity of ethyl acetate MSL extracts against carbapenem reistant pathogens isolated from burn infection. Values represented as presented as mean ± SD, (n=6)

Without the evaluation of antibiofilm potentiality, only MIC determination was not noteworthy because of biofilms microorganism extracellular polysaccharides matrix resulting into microbe strong attachment to biotic/abiotic surfaces which trigger the efflux pumps to expel the drugs or lowering the drugs penetration into the cell [65,66]. Consequently make those drugs molecules resistant to those bacterial strain communities and can spread this resistance genetic material among communities other members. As per past record of *E. coli, K. pneumoniae, P. aeruginosa,* and *S. aureus* microbes ability to form biofilm [67], we had been utilized these carbapenem resistant pathogens isolated from burn wound infections to divulge the antibiofilm activity of our studies prominent ethyl acetate MSL extracts. These extract at the concentration of half of MIC inhibited a little biofilm formation but in MIC concentration it inhibited 50% biofilm formation occurred by *E. coli.* and inhibited less than 50% for other pathogens (Fig. 4). However, it prevented about 80% of biofilm formation in gram-negative *P. aeruginosa* and *E. coli* at 2×MIC concentration which in line with this extracts earlier findings about robust efficiency against those gram staining bacteria, even though it was around 60% in *S. aureus* and *K. pneumoniae*. As comparison to MIC concentrations of MSL, isolated asimicin molecules from *Annona senegalensis* seeds visualized almost comparable biofilm eradication of 36.5 and 43.2% with *S. aureus* and *E. coli* [68]. Contrariwise, methanolic *Carum copticum* extract demonstrated 25 – 60% biofilm inhibition with gram negative (*E. coli, K. pneumoniae, P. aeruginosa* and *A. baumannii*) and 20 – 30% inhibition in gram-positive bacteria (*S. aureus* and *B. cereus*) at current investigations comparable 6.25 mg/ml concentrations level [69]. These superior ability of MSL plant extracts to prevent the formation of biofilms might have been associated to intervention with organic and inorganic nutrients, as well as sedimentation, brownian, electrostatic and Van der Waals interaction factors that promote the bacterial cell growth and adhesion to surfaces [70,71]. The distinctiveness of the present research is that there have been no reports on employing MS to suppress the cell growth of bacterial biofilms yet.

### 3.6 Membrane stabilization potential of MSL and MSS extractives

**Fig. 5:**
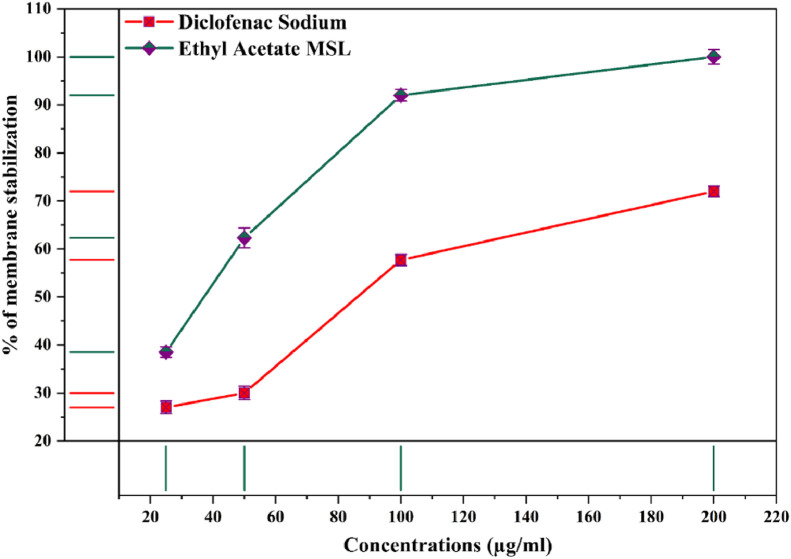
Membrane stabilization potency of ethyl acetate MSL extracts. Values of stabilization are presented as mean ± SD, (n=6)

Through the activation of lysosomal enzymes or inflammatory cytokines in stimulus to wounds, infection, or other factors (hypotonic solution) instigates one of the complex biological states termed as inflammation. This leads to engorged cell membranes, one that cause vasodilation and increased permeability of blood vessels, allowing neutrophils, fluids, and plasma protein to extravasate into the tissues along with hemolysis and hemoglobin oxidation [72,73]. Human red blood cell (HRBC) or erythrocyte membranes are nearly identical to lysosomal membranes [74] and were utilized in stabilization assays to ascertain the anti-inflammatory effects of ongoing research most prominent ethyl acetate MSL extracts ahead of addressing the issues likely arterial hypertension, stroke, heart failure, acute myocardial infarction and renal failure originated by conventional NSAIDs [75]. Ethyl acetate extracts illustrated dose dependent membrane stabilization at the concentrations of 25 – 200 µg/ml (Fig. 5). Of them, at 100 µg/ml concentrations this same extract obtained 92 ± 0.2% membrane stability which explicitly paramount than standard diclofenac sodiums 57.7 ± 0.2% stability at the similar dose level. Tantary et al. (2017), Umukoro and Ashrobi (2006) carried out identical research and figured that the plant extracts they inspected attenuated RBC hemolysis by 70–90% [76,77]. By preventing the expulsion of certain protease and bactericidal enzymes like activated neutrophils lysosomal components, these plant extracts perhaps capable of stabilizing lysosomal membranes or controlling key pro-inflammatory cytokines. Numerous reports have proposed that the presence of flavonoids, terpenoids, and saponin in plants contributes significantly to the usefulness of lysosomal membrane stability [78], as well as being consistent with our phytochemical screening investigations. This is clarified by the fact that Acheflan and Daflon are anti-inflammatory pharmaceutical flavonoid medicines with minimal side effects [72].

### 3.7 Molecular docking simulation of MSL antimicrobial and antiiflammatory robustness

**Fig. 6:**
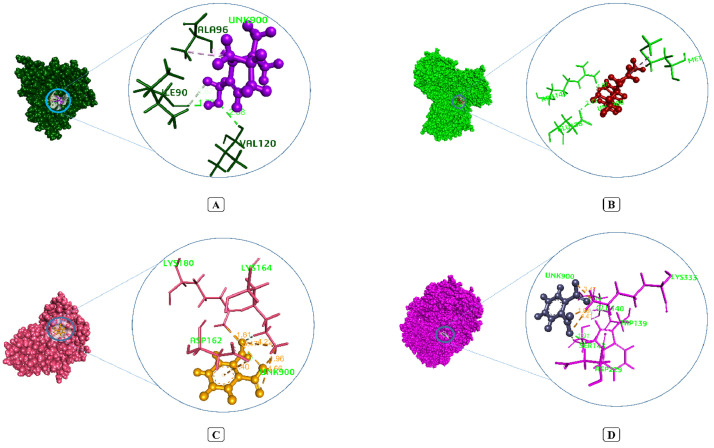
Interacting pose of MSL compounds best binding affinity with corresponding antimicrobial and anti-inflammatory target proteins. (A) 1KZN – Myrtenol, (B) 2VES - T-cardinol, (C) 7BLY - 1,2-Benzenedicarboxylic acid, and (D) 6COX - 1,2-Benzenedicarboxylic acid complex.

Conducting an encyclopedic literature review to analyze 23 chemicals from MSL (Table 8), 22 of them (except Tetratetracontane) had successfully navigated possible bioavailability issues, per the rules of five taken from the 90th percentile of therapeutic candidates that advanced phase II clinical trials [79]. Because the therapeutic success of the relevant pharmacological candidates is contingent on retaining a sufficient dosage in the target location [80]. Subsequently, this therapeutic interaction for effectiveness were virtually verified employing molecular docking tools comprehensively with pathogenic organisms virulent target protein namely DNA gyrase subunit b S. aureus (PDB ID: 1KZN), LpxC of P. aeruginosa (PDB ID: 2VES), Structure of the chitin deacetylase AngCDA from Aspergillus niger (PDB ID: 7BLY) and cyclooxygenase-2 (6COX). Whereby, 1KZN-Myrtenol forming complex with three hydrogen (Val 120, Ile 90) and one hydrophobic bonds of Ala 96 residues generating lower most energy of -5.277 Kcal/mol backed by alpha-cubebene and gamma-Muurolene. This fortify affinity meanwhile conspicuously superior than contemporary drug ciprofloxacins -3.912 Kcal/mol bonding energy (Fig. 6A). Table 9 showed that UDP-3-O-(R-3-hydroxymyristoyl)-N-acetylglucosamine deacetylase (PDB ID: 2VES) similar sturdiest inhibition markedly prominent with T-cadinol (-4.118 Kcal/mol) constituting two H-bond (Arg 143, Glu 138) and one H-phobic (Met 162) interactions that over than reference drugs affinity (Fig. 6B). In terms of antifungal and membrane stabilization efficacy, 1, 2-Benzenedicarboxylic acid pointedly outdated the both sertaconazole and diclofenac sodium interacting energy which were furthest. Formation of three hydrogen bonds of Lys 164 and Lys 180, and six hydrophobic bonds of Lys 164, Lys 180 and Asp 162 residues (Fig. 6C) combining 1,2-Benzenedicarboxylic acid with chitin deacetylase (PDB ID: 7BLY) aiming -5.257 Kcal/mol energy whilst sertaconazole generated -4.011 Kcal/mol. Antiinflammatory mechanism therefore affirmed through quantifying modulation of cyclooxygenase-2 (PDB ID: 6COX) in which 1,2-Benzenedicarboxylic acid displayed excellent binding affinity of -6.797 Kcal/mol with originating six hydrogen and two electrostatic bonds (Fig. 6D). Details binding interaction distinctive features inscribed in Table 10. This following adjacent binding affinity in active site formulated a number variable non-covalent bonds that might be attributed to the possible inhibition of target proteins virulence factors [81]. Literature thereby dissected antimicrobial and fungicide potentialities of one key compound 1, 2-Benzenedicarboxylic acid derivatives isolated from plants [82,83].

**Table 8:** Pharmacokinetic properties of 23 compounds from MSL

Compounds	PubCHem CID	MW≤500	nHA≤10	nHD≤5	MlogP≤5	TPSA	GI absorption	nViolations	Bioavailability
O-Decylhydroxylamine	34704	173.3	2	1	2.72	35.25	High	0	0.55
Myrtenol	10582	152.23	1	1	2.3	20.23	High	0	0.55
Dodecamethyl cyclohexasiloxane	10911	444.92	6	0	-1.28	55.38	High	0	0.55
Alpha-Cubebene	442359	204.35	0	0	5.65	0	Low	1	0.55
Alpha-Copaene	12303902	204.35	0	0	5.65	0	Low	1	0.55
beta-Caryophyllene	5281515	204.35	0	0	4.63	0	Low	1	0.55
1H-cyclopropa[a]naphthalene	12343165	140.18	0	0	4.16	0	Low	1	0.55
cis-beta-Farnesene	5317319	204.35	0	0	4.84	0	Low	1	0.55
gamma-Muurolene	12313020	204.35	0	0	4.63	0	Low	1	0.55
delta-Cadinene	441005	204.35	0	0	4.63	0	Low	1	0.55
Caryophyllene oxide	1742210	220.35	1	0	3.67	12.53	High	0	0.55
2-Pentadecanone, 6,10,14-trimethyl	10408	268.48	1	0	17.07	4.79	High	1	0.55
1,2-Benzenedicarboxylic acid	1017	166.13	4	2	74.6	1.2	High	0	0.55
Humulene epoxide-II	10704181	220.35	1	0	3.56	12.53	High	0	0.55
beta-Himachalene	11586487	204.35	0	0	4.63	0	Low	1	0.55
T-Cadinol	160799	222.37	1	1	3.67	20.23	High	0	0.55
T-Muurolol	3084331	222.37	1	1	3.67	20.23	High	0	0.55
beta-Farnesene	5281517	204.35	0	0	4.84	0	Low	1	0.55
Tonalide	89440	258.4	1	0	4.1	17.07	High	0	0.55
Nonadecane	12401	268.52	0	0	7.15	0	Low	1	0.55
Phthalic acid, butyl hexyl ester	526381	306.4	4	0	3.91	52.6	High	0	0.55
(-)-Spathulenol	13854255	220.35	1	1	3.67	20.23	High	0	0.55
Tetratetracontane	23494	619.19	0	0	0	11.92	Low	2	0.17

**Table 9:** Molecular docking affinity of MSL compounds with antimicrobial and anti-inflammatory targets.

Compounds	1KZN	2VES	7BLY	6COX
O-Decylhydroxylamine	2.316	3.31	2.078	1.192
Myrtenol	**-5.277**	-3.86	-3.297	-4.639
Dodecamethyl cyclohexasiloxane	-2.118	-2.494	-2.458	-1.851
Alpha-Cubebene	-4.897	-3.512	-2.711	-4.105
Alpha-Copaene	-4.283	-3.652	-2.501	-4.593
beta-Caryophyllene	-3.245	-2.525	-2.635	-4.848
1H-cyclopropa[a]naphthalene	-4.462	-3.906	-4.176	-4.322
cis-beta-Farnesene	0.616	1.081	1.491	0.718
gamma-Muurolene	-4.698	-3.462	-3.553	-4.172
delta-Cadinene	-4.045	-3.549	-3.972	-4.643
Caryophyllene oxide	-3.943	-	-	-5.683
2-Pentadecanone, 6,10,14-trimethyl	0.199	1.143	0.362	-2.389
1,2-Benzenedicarboxylic acid	-3.676	-3.926	**-5.257**	**-6.797**
Humulene epoxide-II	-3.214	-3.104	-2.844	-4.334
beta-Himachalene	-4.256	-3.218	-2.462	-4.885
T-Cadinol	-4.69	**-4.118**	-3.084	-5.906
T-Muurolol	-4.124	-4.028	-3.218	-5.119
beta-Farnesene		1.45	1.241	-0.014
Tonalide	-3.551	-3.891	-2.506	-5.706
Nonadecane	2.496	2.954	-	1.378
Phthalic acid, butyl hexyl ester	-2.212	-1.99	-2.127	-3.931
(-)-Spathulenol	-3.346	-3.407	-3.486	-5.466
Ciprofloxacin/ Sertaconazol/ Diclofenac Sodium	-3.912	-3.858	-4.011	-5.983

**Table 10:** Binding interaction of MSL best compounds with antimicrobial and anti-inflammatory target proteins

Ligand-Protein complex	Binding Interaction					
	Hydrogen bond	Distance (Å)	Hydrophobic interactions	Distance (Å)	Electrostatic	Distance (Å)
1KZN - Myrtenol	Val 120	2.57	Ala 96	5.21	-	-
	Ile 90	1.77	-	-		
	Ile 90	2.86				
2VES - T-cardinol	Arg 143	1.86	Met 162	5.41	-	-
	Glu 138	2.12	-	-		
7BLY - 1,2-Benzenedicarboxylic acid	Lys 164	1.95	-	-	Lys 164	1.95
	Lys 164	2.73			Lys 164	4.21
	Lys 180	1.61			Lys 164	4.66
	-	-			Lys 180	1.61
					Lys 180	5.47
					Asp 162	4.4
6COX - 1,2-Benzenedicarboxylic acid	Lys 33	1.86	-	-	Lys 33	3.34
	Lys 33	1.74			Lys 33	4.41
	Trp 139	1.91			-	-
	Trp 139	2.39				
	Gln 241	1.92				
	Ser 143	3.02				

### 3.8 Conclusion

This conclusive study significantly conveyed that the ethyl acetate extract of *Mikania scandens* leaves disclosed broad spectrum antimicrobial properties in multitude parameters of bactericidal, fungicidal and biofilm inhibition against human pathogenic and carbapenem resistant pathogens over stem parts efficacy. Therefore membrane stabilizing modulatory effects in acute inflammation bring forth substantial vascular alterations like anti-inflammatory potency. Furthermore molecular docking assay vindicated those experimental findings precisely. These all were homologous with its traditional grandeur. Since there are a growing number bacterial strains that are resistant to conventional antibiotics, this property may be a helpful ally in the development of medications to combat this culminating microbial resistance problem. Hence, extensive mechanistic function research using these plant extract is still required to invent novel antimicrobial agents for complementary medicine.

## Funding

No particular grant was given to this research by funding organizations in the public, private, or not-for-profit sectors.

## CRediT authorship contribution statement

**Nadia Islam Tumpa:** Data curation, Methodology, Visualization, Investigation, Writing - original draft preparation. **Ankhy Alamgir Asma:** Visualization, Investigation, Writing- Original draft preparation. **Md. Helal Uddin Chowdhury:** Conceptualization, Computational Software, Writing- review & editing, Validation, supervision

## CRediT authorship contribution statement

**Nadia Islam Tumpa:** Data curation, Methodology, Visualization, Investigation, Writing – original draft. **Md. Helal Uddin Chowdhury:** Visualization, Investigation, Writing – original draft. **Ankhy Alamgir Asma:** Conceptualization, Software, Writing – review & editing, Validation, Supervision.

## Declaration of Competing Interest

No conflicts of interest.

## Data Availability

Data will be made available on request.
